# Supplementary material for: Dynamic networks observed in the nucleosome core particles couple the histone globular domains with DNA
Source: Commun Biol. 2020 Oct 30;3:639. doi: 10.1038/s42003-020-01369-3 (PMC7599221; doi:10.1038/s42003-020-01369-3)
Supplement: Supplementary file 1 — Supplementary Information [file 42003_2020_1369_MOESM1_ESM.pdf]

Dynamic Networks Observed in the Nucleosome Core Particles Couple the Histone Globular Domains with DNA

Xiangyan Shi[1*+], Chinmayi Prasanna[2+], Aghil Soman[2], Konstantin Pervushin[2] and Lars Nordenskiöld[2*]

1. School of Physical and Mathematical Sciences, Nanyang Technological University, 21 Nanyang Link, Singapore 637371

2. School of Biological Sciences, Nanyang Technological University, 60 Nanyang Drive, Singapore 637551

+ Those authors contributed equally

*For correspondence: shi.xiangyan@ntu.edu.sg (XS); larsnor@ntu.edu.sg (LN)

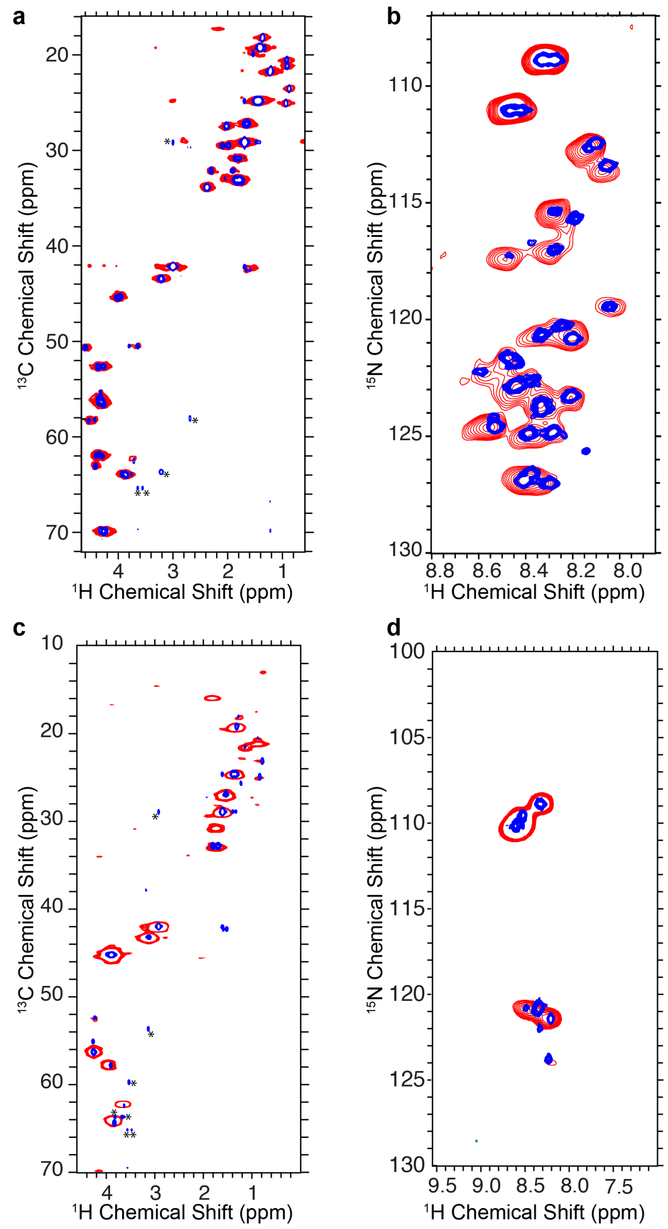

Supplementary Fig. 1 **a** Overlaid SSNMR ^{1}H-^{13}C correlation spectrum of the precipitated Widom 601 NCP (red) and liquid-state HSQC NMR spectrum of the solubilized NCP (blue) contained uniformly ^{13}C, ^{15}N labelled hH3. **b** Overlaid SSNMR ^{1}H-^{15}N correlation spectrum of the precipitated Widom 601 NCP (red) and liquid-state HSQC NMR spectrum of the solubilized NCP (blue) contained uniformly ^{13}C, ^{15}N labelled hH3. **c** Overlaid SSNMR ^{1}H-^{13}C correlation spectrum of the precipitated Widom 601 NCP (red) and liquid-state HSQC NMR spectrum of the solubilized NCP (blue) contained uniformly ^{13}C, ^{15}N labelled hH4. **d** Overlaid SSNMR ^{1}H-^{15}N correlation spectrum of the precipitated Widom 601 NCP (red) and liquid-state HSQC NMR spectrum of the solubilized NCP (blue) contained uniformly ^{13}C, ^{15}N labelled hH4. Asterisks mark the peaks do not belong to the NCPs.

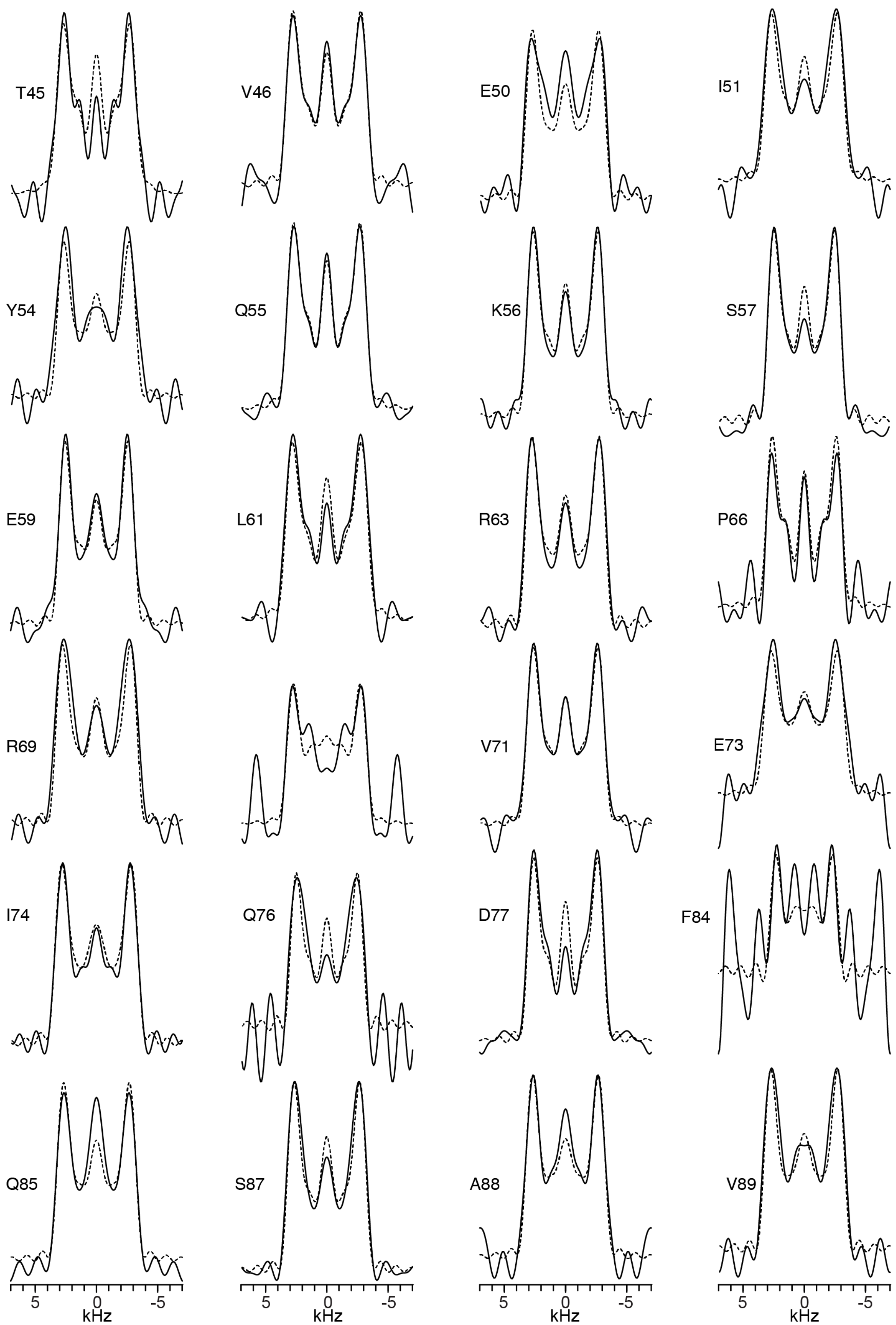

Supplementary Fig. 2 Site-resolved experimental (solid) and simulated (dotted) ^{1}H-$^{13}C\alpha$ dipolar line shapes for hH3 in the Widom 601 NCP. The experimental line shapes are extracted from a 3D DIPSHIFT with $R12_1^4$ symmetry dipolar recoupling pulses. The SIMPSON output values of the root-mean-square (rms) deviation between experimental and simulated line shapes are provided.

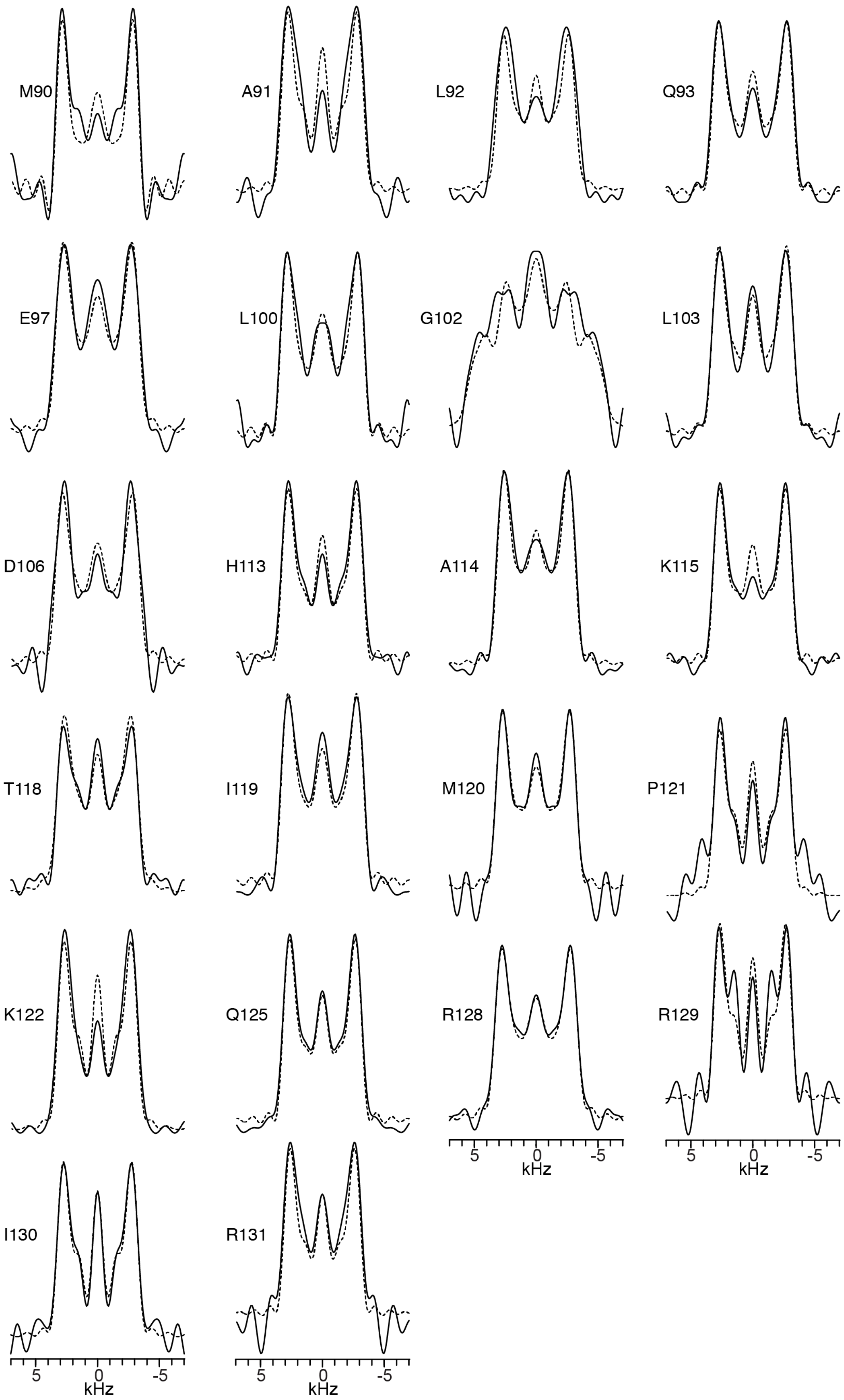

Supplementary Fig. 2 (Continued)

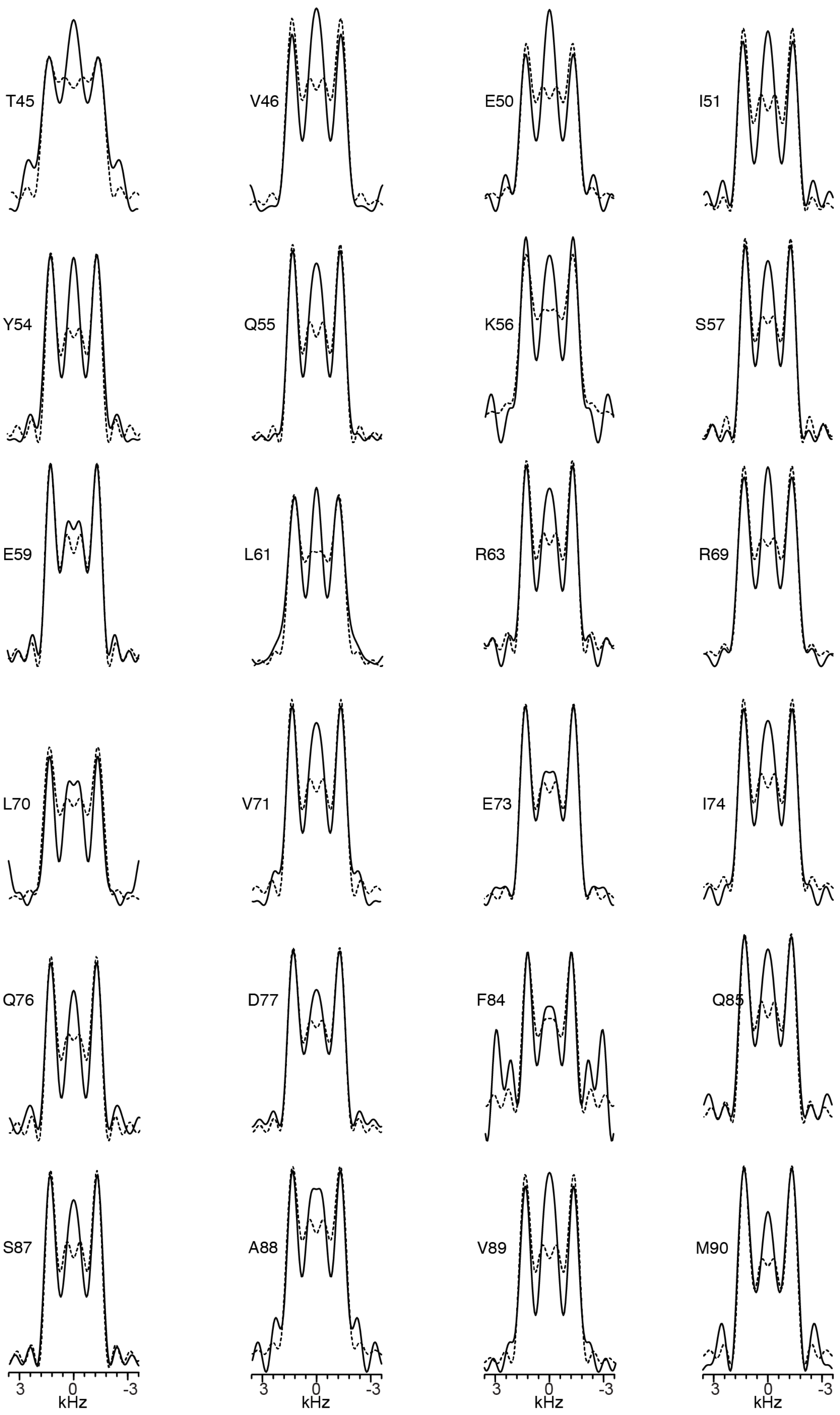

Supplementary Fig. 3 Site-resolved experimental (solid) and simulated (dotted) ^{1}H-^{15}N dipolar line shapes for hH3 in the Widom 601 NCP. The experimental line shapes are extracted from a 3D DIPSHIFT with $R12_1^4$ symmetry dipolar recoupling pulses. The SIMPSON output values of the root-mean-square (rms) deviation between experimental and simulated line shapes are provided.

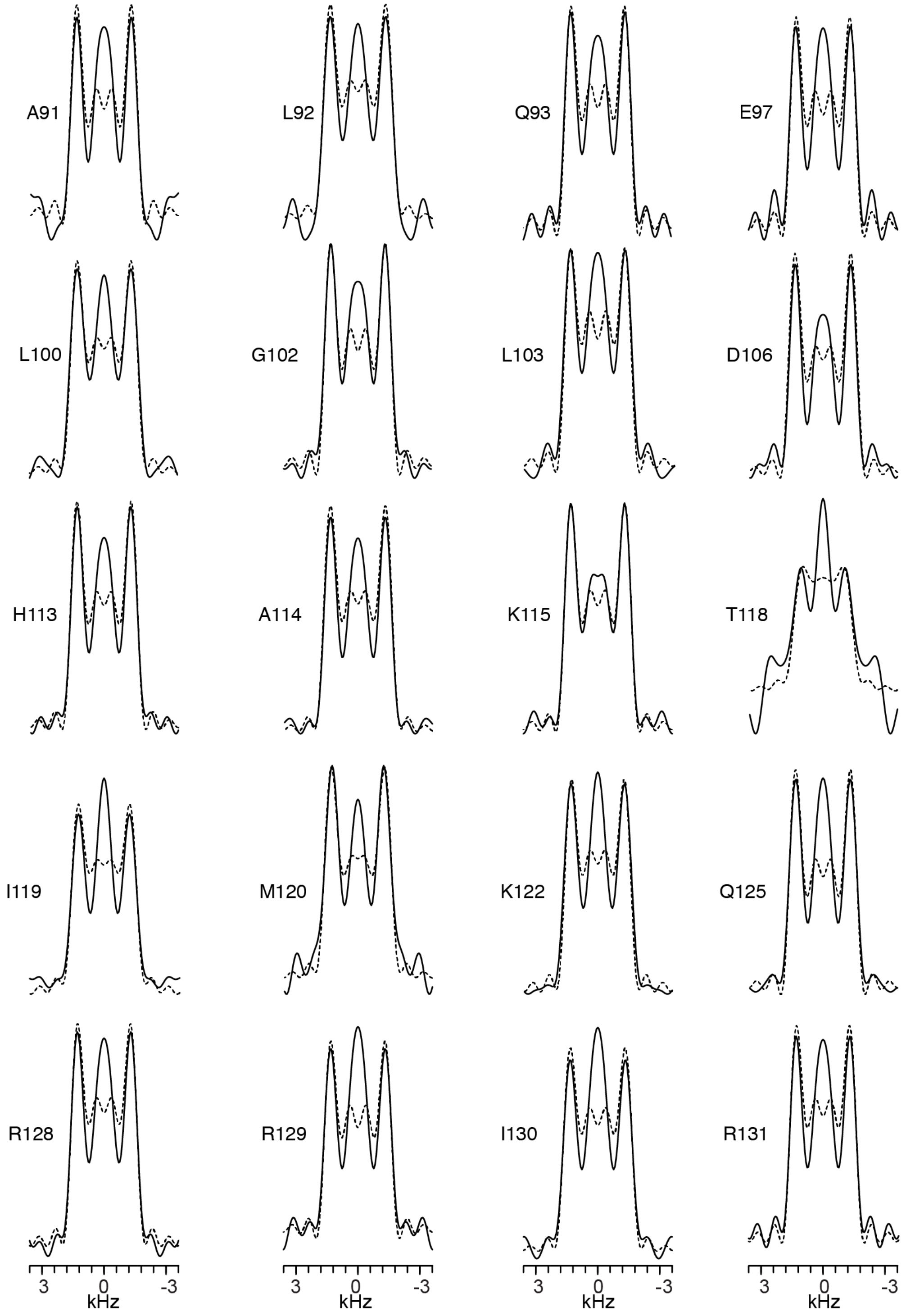

Supplementary Fig. 3 (Continued)

Supplementary Table 1 SSNMR parameters of experiments performed for the Widom 601 and telomeric NCPs.

**Field (T)**	18.8						
**Sample**	Widom 601 NCP (^{13}C, ^{15}N hH3)				Telomeric NCP (^{13}C, ^{15}N hH3)		
**weight**	55.2 mg				11.7 mg		
**hydration level**	65% (by wt)				66% (by wt)		
**Experiment**	CC	NCA	NCO	CANCO	CC	NCA	NCO
**MAS rate (kHz)**	15.151	15.151	15.151	15.151	15.151	15.151	15.151
**Probe**	3.2 mm HCN EFree MAS probe				1.9 mm HCN MAS probe		
**transfer 1**	HC CP	HN CP	HN CP	HC CP	HC CP	HN CP	HN CP
rf field (kHz), ^{1}H	70.4	52.2	52.2	70.0	67.4	64.9	64.9
shape	ramp	ramp	ramp	ramp	ramp	ramp	ramp
rf field (kHz), ^{15}N/^{13}C	53.0	37.9	37.9	53.0	53.0	53.0	53.0
transfer time	1.0	1.0	1.0	0.8	1.0	1.2	1.2
carrier (ppm)	103.0	–	–	55.0	–	–	–
^{13}C,^{15}N	–	115.4	115.4	–	102.7	120.1	120.1
**transfer 2**	DARR	NCA SPECIFIC	NCO SPECIFIC	CAN SPECIFIC	DARR	NCA SPECIFIC	NCO SPECIFIC
rf field (kHz), ^{13}C	–	28.5	45.1	28.9	–	27.2	43.9
shape		tangent	tangent	tangent		tangent	tangent
rf field (kHz), ^{15}N	–	37.9	22.7	37.9	–	37.9	22.7
rf field (kHz), ^{1}H cw	14.1	89.4	89.4	89.4	15.1	93.6	91.8
transfer time	20	4.0	4.0	3.0	20	3.0	3.5
carrier (ppm)	103.0	58.0	178.0	55.0	102.7	54.7	177.8
^{13}C,^{15}N	–	115.4	115.4	115.4	–	120.1	120.1
**transfer 3**	–	–	–	NCO SPECIFIC	–	–	–
rf field (kHz), ^{13}C<br>shape	–	–	–	45.0	–	–	–
rf field (kHz), ^{15}N	–	–	–	22.7	–	–	–
rf field (kHz), ^{1}H cw	–	–	–	89.4	–	–	–
transfer time	–	–	–	3.0	–	–	–
carrier (ppm)	–	–	–	178.0	–	–	–
^{13}C,^{15}N	–	–	–	115.4	–	–	–
**digitalization, F1**	C	N	N	CA	C	N	N
t1 increments	768	192	192	84	768	76	76
sweep width (kHz)	45453	7575.5	7575.5	7575.5	45453	3030.2	3030.2
acquisition time (ms)	8.4	12.7	12.7	5.5	8.4	12.5	12.5
**digitalization, F2**	C	C	C	N	C	C	C
t2 increments	1536	832	832	48	1536	448	448
sweep width (kHz)	53571.43	29761.904	29761.904	3030.2	53571.43	15151.516	15151.516
acquisition time (ms)	14.4	14.0	14.0	7.9	14.4	14.8	14.8
**digitalization, F3**	–	–	–	CO	–	–	–
t3 increments	–	–	–	1088	–	–	–
sweep width (kHz)	–	–	–	45454.547	–	–	–
acquisition time (ms)	–	–	–	11.9	–	–	–
**^{1}H decoupling<br>rf filed (kHz)<br>shape**	73.5<br>SPINAL 64	73.5<br>SPINAL 64	73.5<br>SPINAL 64	73.5<br>SPINAL 64	81.4<br>SPINAL 64	81.4<br>SPINAL 64	81.4<br>SPINAL 64
**number of scans**	96	192	196	56	512	4224	5832
**pulse delay (s)**	1.5	1.5	1.5	1.5	1.5	1.5	1.5

Supplementary Table 1 (Continued)

**Field (T)**	18.8					
**Sample**<br>**weight**<br>**hydration level**	Widom 601 NCP (^{13}C, ^{15}N hH4)<br>46 mg<br>57% (by wt)			Telomeric NCP (^{13}C, ^{15}N hH4)<br>13.7 mg<br>63% (by wt)		
**Experiment**	CC	NCA	NCO	CC	NCA	NCO
**MAS rate (kHz)**	15.151	15.151	15.151	17.857	17.857	17.857
**Probe**	3.2 mm HCN EFree MAS probe			1.9 mm HCN MAS probe		
**transfer 1**	HC CP	HN CP	HN CP	HC CP	HN CP	HN CP
rf field (kHz), ^{1}H<br>shape	82.2<br>ramp	63.7<br>ramp	63.7<br>ramp	81.5<br>ramp	76.9<br>ramp	76.9<br>ramp
rf field (kHz), ^{15}N/^{13}C	56.9	44.6	44.6	62.5	44.6	44.6
transfer time	1.4	1.4	1.4	1.2	1.0	1.0
carrier (ppm)<br>^{13}C, ^{15}N	103.5<br>–	–<br>115.8	–<br>115.8	–<br>102.6	–<br>115.0	–<br>115.0
**transfer 2**	DARR	NCA SPECIFIC	NCO SPECIFIC	DARR	NCA SPECIFIC	NCO SPECIFIC
rf field (kHz), ^{13}C<br>shape	–	30.8,<br>tangent	48.8,<br>tangent	–	31.7<br>tangent	51.8<br>tangent
rf field (kHz), ^{15}N	–	44.6	26.8	–	44.6	26.8
rf field (kHz), ^{1}H cw	15.5	88.6	90.6	16.1	93.6	91.8
transfer time	20	3.5	4.5	20	3.5	4.0
carrier (ppm)<br>^{13}C, ^{15}N	103.5<br>–	55.5<br>115.8	178.5<br>115.8	102.6<br>–	54.6<br>115.0	177.6<br>118
**transfer 3**	–	–	–	–	–	–
rf field (kHz), ^{13}C<br>shape	–	–	–	–	–	–
rf field (kHz), ^{15}N	–	–	–	–	–	–
rf field (kHz), ^{1}H cw	–	–	–	–	–	–
transfer time	–	–	–	–	–	–
carrier (ppm)<br>^{13}C, ^{15}N	–<br>–	–<br>–	–<br>–	–<br>–	–<br>–	–<br>–
**digitalization, F1**	C	N	N	C	N	N
t1 increments	896	112	112	896	88	72
sweep width (kHz)	53571	4464.25	4464.25	53571	3571.4	2976.167
acquisition time (ms)	8.4	12.5	12.5	8.4	12.3	12.1
**digitalization, F2**	C	C	C	C	C	C
t2 increments	1664	512	512	1664	1536	512
sweep width (kHz)	53571.43	17857.143	17857.143	53571.43	53571.43	17857.143
acquisition time (ms)	15.5	14.3	14.3	15.5	14.3	14.3
**digitalization, F3**	–	–	–	–	–	–
t3 increments	–	–	–	–	–	–
sweep width (kHz)	–	–	–	–	–	–
acquisition time (ms)	–	–	–	–	–	–
**^{1}H decoupling**<br>**rf filed (kHz)**<br>**shape**	80.6<br>SPINAL 64	80.6<br>SPINAL 64	80.6<br>SPINAL 64	78.5<br>SPINAL 64	78.5<br>SPINAL 64	78.5<br>SPINAL 64
**number of scans**	48	196	196	168	1728	1408
**pulse delay (s)**	1.5	1.5	1.5	1.5	1.5	1.5
